# Associations among political voting preference, high-risk health status, and preventative behaviors for COVID-19

**DOI:** 10.1186/s12889-022-12633-y

**Published:** 2022-02-04

**Authors:** Thalia Porteny, Laura Corlin, Jennifer D. Allen, Kyle Monahan, Andrea Acevedo, Thomas J. Stopka, Peter Levine, Keren Ladin

**Affiliations:** 1 Department of Community Health, Tufts University, 574 Boston Avenue, Suite 118G, Medford, MA 02155 USA; 2 Department of Occupational Therapy, Tufts University, 574 Boston Avenue, Suite 118G, Medford, MA 02155 USA; 3 Department of Public Health and Community Medicine, Tufts University School of Medicine, 136 Harrison Ave, Boston, MA 02111 USA; 4 Department of Civil and Environmental Engineering, Tufts University School of Engineering, 200 College Ave, Medford, MA 02155 USA; 5 Data Lab, Tufts Technology Services, Tufts University, 16 Dearborn Road, Medford, MA 02155 USA; 6 Jonathan M. Tisch College of Civic Life, Tufts University, 163 Packard Ave, Medford, MA USA

**Keywords:** COVID-19, Preventative behaviors, Underlying health conditions, Political voting preference

## Abstract

**Background:**

We investigate the relationships among political preferences, risk for COVID-19 complications, and complying with preventative behaviors, such as social distancing, quarantine, and vaccination, as they remain incompletely understood. Since those with underlying health conditions have the highest mortality risk, prevention strategies targeting them and their caretakers effectively can save lives. Understanding caretakers’ adherence is also crucial as their behavior affects the probability of transmission and quality of care, but is understudied. Examining the degree to which adherence to prevention measures within these populations is affected by their health status vs. voting preference, a key predictor of preventative behavior in the U. S, is imperative to improve targeted public health messaging. Knowledge of these associations could inform targeted COVID-19 campaigns to improve adherence for those at risk for severe consequences.

**Methods:**

We conducted a nationally-representative online survey of U.S. adults between May–June 2020 assessing: 1) attempts to socially-distance; 2) willingness/ability to self-quarantine; and 3) intention of COVID-19 vaccination. We estimated the relationships between 1) political preferences 2) underlying health status, and 3) being a caretaker to someone with high-risk conditions and each dependent variable. Sensitivity analyses examined the associations between political preference and dependent variables among participants with high-risk conditions and/or obesity.

**Results:**

Among 908 participants, 75.2% engaged in social-distancing, 94.4% were willing/able to self-quarantine, and 60.1% intended to get vaccinated. Compared to participants intending to vote for Biden, participants who intended to vote for Trump were significantly less likely to have tried to socially-distance, self-quarantine, or intend to be vaccinated. We observed the same trends in analyses restricted to participants with underlying health conditions and their caretakers Underlying health status was independently associated with social distancing among individuals with obesity and another high-risk condition, but not other outcomes.

**Conclusion:**

Engagement in preventative behavior is associated with political voting preference and not individual risk of severe COVID-19 or being a caretaker of a high-risk individual. Community based strategies and public health messaging should be tailored to individuals based on political preferences especially for those with obesity and other high-risk conditions. Efforts must be accompanied by broader public policy.

## Introduction

In the United States, up to 92.8% of COVID-19 deaths are found to be associated with a pre-existing comorbidity [1]. Mortality from COVID-19 has thus disproportionately affected people with co-morbid conditions such as cardiovascular disease, diabetes, chronic respiratory disease, hypertension, and obesity [2, 3]. Now that safe and effective vaccines are available, those with high-risk conditions are a priority in most state vaccination programs [4]. Vaccination program success, partly defined by lives saved among individuals with co-morbid conditions, depends on people’s willingness to be vaccinated [5, 6]. Yet, concerns about new variants’ (e.g., omicron) ability to circumvent vaccine protection point to a need for continued adherence to mitigation strategies like social distancing and mask wearing [7–10].

Few studies have assessed adherence to COVID-19 prevention measures among populations at high risk of developing severe COVID-19 infection due to comorbidities. Some studies published early in the pandemic (i.e., before June 2020) focused only on specific cities or other small regions [11, 12], or focused on populations outside of the United States [13–18]. Findings suggest that individuals with underlying health conditions (e.g., cardiometabolic diseases) might be more likely to adhere to measures like social distancing [19] and might be more likely to intend to be vaccinated against COVID-19 because of their increased vulnerability [14, 20]. Other research did not observe this trend and found that high-risk conditions that are highly prevalent in the U.S. population such as being obese, were not significant predictors of preventative behaviors [21]. Moreover, some vulnerable people did not believe they were at an increased risk of complications and/or did not behave differently than those without underlying conditions [13, 15]. The mixed evidence points to a critical gap in our understanding of factors that might increase adherence to mitigation strategies among high-risk populations and their caretakers [22]. Caretakers’ adherence is particularly relevant because their behavior affects the probability of transmission to a vulnerable population and because they would not be able to provide care should they contract COVID-19, but there is limited research on prevention adherence or public policy guidance that targets this group [23]. One reason that evidence is lacking is that many studies that assessed adherence to COVID-19 prevention measures were ecologic in nature (i.e., they did not include individual-level health or behavior data) [21, 24, 25].

Political affiliation is one notable predictor of individuals’ intention to get vaccinated against COVID-19 [26] and to practice social distancing during the COVID-19 pandemic, but these studies are not stratified by people’s underlying health conditions or COVID-19 risk [27–32]. Differences in willingness to be vaccinated for other vaccine-preventable infectious diseases associated with political affiliation predates the COVID pandemic [33]. For example, Republicans were significantly less likely than Democrats to be vaccinated for the H1N1 influenza vaccine [34]. Using voter preferences as a proxy for political affiliation, researchers have found similar trends; counties with lower proportions of people who voted for Trump in the 2016 election were more responsive to stay-at-home orders and other COVID-19 lockdown mandates [21]. Additionally, individuals who typically refused some or all vaccines were more likely to support Donald J. Trump over Joseph R. Biden or other candidates [33, 35]. One possible explanation for substantial partisan gaps in adherence to social distancing are beliefs about the severity of COVID-19 and the importance of the preventative behavior [32]. A theoretical reason for this divide is that Republicans are more likely than other voters to believe in conspiracy theories regarding the COVID-19 virus and vaccination and are therefore less likely to adhere to measures [36–38].

Nevertheless, gaps remain in understanding the associations between individuals’ voter preferences, their underlying health status, and their adherence to COVID-19 prevention measures. Since those with underlying health conditions have the highest mortality risk, prevention strategies that target them and their loved ones effectively can help maximize lives saved and avert some social and economic costs of this disease [39]. Examining if adherence to prevention measures within the vulnerable populations is affected by voting preference is imperative to improve targeted public health messaging and inform policy. The information can also help promote adherence to pandemic control measures now and in future health crises. Therefore, we used individual-level survey data from a nationally representative sample to examine whether voting preferences were associated with COVID-19 prevention measures and whether having underlying health conditions or caring for someone who does moderates this relationship. In this exploratory study, we hypothesized that people with underlying health conditions are more likely to adhere to COVID-19 prevention measures, but Republicans with underlying health conditions will adhere less than their Democrat counterparts.

## Methods

We analyzed nationally representative, cross-sectional Tufts Equity in Health, Wealth, and Civic Engagement survey data collected between May 29th and June 10th, 2020 by the social science company Ipsos. Ipsos used KnowledgePanel®, a web-based panel that captures the largest, online, probability-based panel designed to be representative of the non-institutionalized U.S. adult population aged 18 and older [40–42].

### Sample

A sample of 1980 panel members were selected for survey participation in English or Spanish. Sixty-four percent (*n* = 1267) of the invited individuals responded to the Tufts Health, Wealth, and Civic Engagement survey. Members were randomly selected to represent the U.S. population with a measurable level of accuracy, this is a unique characteristic that is usually not featured in panels where participants opt-in [40, 41]. Once members joined the Panel, they were asked to complete a short demographic survey. All respondents were accorded privacy and confidentiality protections. Estimated annual attrition of Panel members is about 18% [43]. Of the 1267 respondents for the Tufts Equity in Health, Wealth, and Civic Engagement survey, we included 908 participants with complete data relating to the primary exposure and outcome variables. The median survey completion time was 17 min. All study protocols were approved by the Tufts University Social, Behavioral, and Educational Research Institutional Review Board (protocol STUDY00000428).

### Measures

We used validated questions from the standardized CoRonavIruS Health Impact Survey (CRISIS) to investigate adherence to COVID-19 preventative measures of interest [44]. We assessed our primary outcomes with three questions: “*Have you tried to isolate yourself from contact with other people because of Coronavirus? (yes or no)”; “If you were advised to self-quarantine (stay at home) for 14 days, would you be willing and able to comply? (yes or no)”;* and *“If a vaccine became available to prevent Coronavirus, would you get it? (yes, no, or don’t know).”* We assessed our primary independent variable of interest “political voting preference” by asking*: “If the election for president were held today, and Donald Trump were the Republican candidate and Joe Biden were the Democratic candidate, for whom would you vote? (Donald Trump, Joe Biden, neither or someone else).”* As a secondary independent variable, “underlying health status,” we developed a dichotomous metric (has/does not have any underlying conditions) indicating whether the participant had one or more underlying health conditions that the CDC designates as increased risk of severe illness from COVID-19. This variable included affirmative self-reported responses to any of the following: smoked ≥100 cigarettes in lifetime, obesity (body mass index [BMI] ≥ 30 kg/m^2^ calculated using self-reported height and weight) [45] or diagnosed by a qualified medical professional with heart attack, heart disease, other heart condition, cancer, kidney disease, asthma, chronic bronchitis, chronic obstructive pulmonary disease, diabetes, or pre-diabetes. As a third independent variable, individuals were classified as “caretakers of someone with an underlying health condition” if they reported being the caretaker of an adult diagnosed by a qualified medical professional with heart attack, heart disease, other heart condition, cancer, kidney disease, asthma, chronic bronchitis, chronic obstructive pulmonary disease, or diabetes [45].

### Analysis

We examined univariate distributions for all variables, as well as bivariate relationships of each independent variable and covariate with each dependent variable. We constructed logistic regression models to estimate associations of independent variables and dichotomous dependent variables (self-isolation and self-quarantine); separate models for each independent variable and dependent variable of interest. We used multinomial logistic regression models to estimate associations of each independent variable of interest with willingness to be vaccinated (separate models for each independent variable of interest). The independent variables of interest included 1) presidential candidate preference (Biden, Trump, or neither/someone else); 2) having high-risk underlying conditions; and 3) caring for someone with high-risk underlying conditions. We used separate models because we were interested in understanding the magnitude and direction in the associations of each exposure and outcome independently. We conducted a sensitivity analysis to examine associations between presidential candidate preference and dependent variables among participants with high risk underlying conditions. Additionally, we conducted a sensitivity analysis to assess whether having obesity (in the absence of other high-risk underlying health conditions), having any high-risk health condition other than obesity, or experiencing both obesity and at least one other high-risk health condition were associated with any of the dependent variables of interest (referent group = not having obesity or any other high-risk health conditions). The rationale for this additional analysis was to assess whether any observed association between having a high-risk underlying condition and our dependent variables was due to the behavior among the obese [46]. All models were adjusted for covariates determined a priori*:* race/ethnicity (Hispanic, non-Hispanic Black, non-Hispanic White, and 2+ races/ethnicities or other), age (continuous), gender (female and male), educational attainment (less than high school, high school, some college, and Bachelor’s degree or higher), and annual household income as a ratio of the 2020 family-sized adjusted Federal Poverty Line [47]. All analyses were conducted in Stata v16 and we applied sample weights to ensure representativeness of the U.S. population [48]. For details on the weighting methods, please refer to Stopka et al. [49]

## Results

Of the 908 participants, 52.8% were female, 64.0% were non-Hispanic White, 31.3% had attained at least a Bachelor’s degree, and the mean age was 50.4 years (95% confidence interval [CI] = 49.0–51.7 years; Table 1). Almost half of our sample (48.9%) reported that they would vote for Biden, 34.4% for Trump, and 16.8% were unsure or undecided. Most (75.2%) respondents had tried to socially distance because of COVID-19, 94.4% were willing/able to self-quarantine if advised to do so, and 60.1% reported that they would get a COVID-19 vaccine.

**Table 1:** Participant characteristics (*n* = 908)^a^

	Socially-distance		Self-quarantine		Intent to get vaccinated			
	No	Yes	No	Yes	No	Yes	Unsure	Total
	% (n)	% (n)	% (n)	% (n)	% (n)	% (n)	% (n)	% (n)
**Total % (n)**	24.8 (212)	75.2 (696)	5.6 (50)	94.4 (858)	15.9 (130)	60.1 (562)	24.0 (216)	100 (908)
**Voting preferences**								
**Biden**	21.0 (81)	79.0 (345)	2.7 (9)	97.3 (417)	10.0 (33)	71.7 (316)	18.3 (77)	48.9 (426)
**Trump**	31.4 (102)	68.6 (244)	10.7 (35)	89.3 (311)	26.1 (82)	47.8 (174)	26.1 (90)	34.4 (346)
**Other/undecided**	22.3 (29)	77.7 (107)	3.6 (6)	96.4 (130)	12.4 (15)	51.4 (72)	36.3 (49)	16.8 (136)
**Health**								
**Caretaker of someone with high-risk underlying health conditions**	19.3 (12)	80.7 (57)	9.1 (6)	90.9 (63)	17.3 (12)	64.9 (45)	17.9 (12)	7.3 (69)
**High-risk underlying health conditions**	25.6 (188)	74.4 (592)	6.0 (46)	94.0 (734)	16.6 (116)	59.0 (475)	24.4 (189)	86.1 (780)
**No high-risk underlying health condition**	21.0 (23)	79.0 (94)	2.5 (3)	97.5 (114)	11.2 (12)	65.0 (78)	23.8 (27)	13.8 (117)
**Obesity (body mass index ≥ 30 kg/m**^**2**^								
	27.8 (99)	72.2 (276)	6.5 (23)	93.5 (352)	20.5 (68)	54.7 (217)	24.8 (90)	43.6 (375)
**Race/Ethnicity**								
**Hispanic**	27.6 (30)	72.4 (82)	4.0 (5)	96.0 (107)	16.1 (19)	61.3 (70)	22.6 (23)	17.4 (112)
**Non-Hispanic Black**	32.1 (27)	67.9 (61)	4.6 (3)	95.4 (85)	27.3 (20)	50.3 (45)	22.5 (23)	12.7 (88)
**Non-Hispanic White**	21.1 (132)	78.9 (514)	6.1 (36)	93.9 (610)	13.9 (83)	61.7 (409)	24.5 (154)	64.0 (646)
**2+ Races/other**	41.3 (23)	58.7 (39)	7.3 (6)	92.7 (56)	13.1 (8)	60.2 (38)	26.7 (16)	5.9 (62)
**Age**								
**18–29**	28.6 (20)	71.4 (49)	6.4 (5)	93.6 (64)	18.2 (12)	64.0 (46)	17.8 (11)	16.1 (69)
**30–44**	25.8 (43)	74.2 (129)	5.9 (11)	94.1 (161)	14.1 (24)	57.3 (101)	28.6 (47)	22.7 (172)
**45–59**	31.4 (78)	68.6 (181)	6.4 (16)	93.6 (243)	22.9 (55)	53.1 (141)	24.0 (63)	27.9 (259)
**60+**	16.8 (71)	83.2 (337)	4.4 (18)	95.6 (390)	10.2 (39)	65.9 (274)	24.0 (95)	33.3 (408)
**Gender**								
**Female**	21.4 (85)	78.6 (370)	4.0 (16)	96.1 (439)	16.5 (66)	55.6 (261)	27.9 (128)	52.8 (455)
**Male**	28.7 (127)	71.3 (326)	7.5 (34)	92.5 (419)	15.2 (64)	65.1 (301)	19.7 (88)	47.2 (453)
**Educational attainment**								
Less than High school	31.0 (21)	69.0 (46)	5.7 (5)	94.3 (62)	25.4 (18)	50.1 (34)	24.5 (15)	10.3 (67)
**High school**	36.8 (92)	63.3 (181)	8.9 (23)	91.1 (250)	22.8 (55)	42.6 (124)	34.6 (94)	29.0 (273)
**Some college**	20.7 (49)	79.3 (203)	6.7 (17)	93.3 (235)	17.0 (40)	61.5 (159)	21.6 (53)	29.4 (252)
**Bachelor’s degree or higher**	15.7 (50)	84.3 (266)	1.5 (5)	98.5 (311)	5.4 (17)	78.2 (245)	16.4 (54)	31.3 (316)
**Ratio of household income to Federal Poverty Line**^b^	3.7 (2.1–6.3)	4.3 (2.5–7.9)	3.1 (1.8–4.6)	4.3 (2.5–7.5)	3.3 (1.9–5.2)	4.6 (2.6–8.0)	3.7 (1.9–6.5)	4.3 (2.5–7.3)

^a^All counts are unweighted and all proportions are weighted. Percentages may not all total 100 due to rounding

^b^The values of the ratio of annual household income to the 2020 family-size adjusted Federal Poverty Line (FPL) are expressed in median (range) and IQR (interquartile range: interval from percentile 25–75)

Compared to participants who reported a preference to vote for Biden, participants who reported a preference for Trump were significantly less likely to have tried to socially-distance (odds ratio (OR) = 0.48, 95% CI = 0.32, 0.73; *p* < 0.001; *F* = 6.59), to be able/willing to self-quarantine (OR = 0.28, 95% CI = 0.13, 0.64; *p* < 0.003; *F* = 3.95), to intend to be vaccinated against COVID-19 (versus not be vaccinated; relative risk ratio (RRR) = 0.15, 95% CI = 0.08, 0.26; *p* < 0.001; *F* = 14.04), and to be unsure about their vaccination intentions (versus intend to not be vaccinated; RRR = 0.34, 95% CI = 0.18, 0.62; *p* < 0.001; *F* = 14.04). There were no significant differences between individuals who indicated a preference to vote for Biden and undecided individuals for any outcome (Table 2). Similarly, having an underlying health condition or caring for someone who does were not associated with any of the adherence measures (Table 2).

**Table 2:** Associations among political affiliation, health status, and COVID-19 prevention behavior

		Social distance			Self-quarantine			Intend to get vaccinated vs. intend to not get vaccinated			Unsure about vaccination intentions vs. intend to not get vaccinated		
Model^**a**^	Comparison	OR^b^	95% CI^b^	F^c^	OR	95% CI	F	RRR^b^	95% CI	F	RRR	95% CI	F
**1:** political affiliation	Trump vs. Biden	0.48***	[0.32, 0.73]	6.59	0.28**	[0.13, 0.64]	5.99	0.15***	[0.08, 0.26]	14.04	0.34***	[0.18, 0.62]	14.04
	Other/neither vs. Biden	1.00	[0.57, 1.75]		0.92	[0.29, 2.89]		0.56	[0.25, 1.24]		1.42	[0.65, 3.10]	
**2:** health status	Underlying health condition vs. not	0.90	[0.52, 1.56]	0.13	0.54	[0.15, 2.00]	0.85	0.86	[0.41, 1.82]	0.24	0.75	[0.32, 1.74]	0.24
**3.** caretaker of someone with underlying health condition	Caretaker of someone with underlying health condition vs. not	1.30	[0.59, 2.84]	0.43	0.40	[0.13, 1.19]	2.72	0.92	[0.39, 2.15]	1.06	0.57	[0.23, 1.39]	1.06
**4:** political affiliation among individuals with underlying health conditions – sensitivity analysis	Trump vs. Biden	0.50**	[0.32, 0.78]	5.87	0.37*	[0.16, 0.85]	3.70	0.14***	[0.07, 0.26]	12.77	0.37***	[0.19, 0.73]	12.77
	Other/neither vs. Biden	1.12	[0.60, 2.10]		0.96	[0.29, 3.15]		0.51	[0.22, 1.20]		1.36	[0.59, 3.15]	
**5:** underlying health – sensitivity analysis	Obesity only vs. neither obesity nor other high-risk health conditions	1.52	[0.54, 4.25]	2.89	0.65	[0.07, 6.57]	0.62	1.27	[0.29, 5.53]	0.69	1.57	[0.31, 7.96]	0.69
	Other high-risk health conditions only vs. neither obesity nor other high-risk health conditions	1.01	[0.58, 1.77]		0.56	[0.15, 2.06]		0.93	[0.43, 1.98]		0.78	[0.33, 1.84]	
	Obesity and other health conditions vs. neither obesity nor other high-risk health conditions	0.48*	[0.24, 0.96]		0.36	[0.08, 1.65]		0.61	[0.24, 1.53]		0.46	[0.16, 1.31]	

^a^All models were adjusted for race/ethnicity (Hispanic, non-Hispanic Black, non-Hispanic White, and 2+ races/ethnicities or other), age (continuous), gender (female and male), educational attainment (less than high school, high school, some college, and Bachelor’s degree or higher), and income as a ratio of the 2020 family-sized adjusted Federal Poverty Line

^b^*OR* Odds ratio, *CI* Confidence interval, *RRR* = Relative risk ratio with interpretation equivalent to odds ratios in logistic regression

^c^F-statistic = Chi-square Adjusted Wald Test were conducted for each model

*p < .05 **p < .01 ***p < .001

In a sensitivity analysis including only individuals with underlying health conditions, a preference to vote for Trump (versus Biden) was significantly associated with a lower odds of having tried to socially distance (OR = 0.50, 95% CI = 0.32, 0.78; *p* < 0.002; *F* = 4.40), being able/willing to self-quarantine (OR = 0.37, 95% CI = 0.16, 0.85; *p* < 0.025; F-statistic = 3.70), intending to be vaccinated against COVID-19 (versus intending to not be vaccinated; RRR = 0.14, 95% CI = 0.07, 0.26; *p* < 0.001; *F* = 12.77), and being unsure about vaccination intentions (versus intending to not be vaccinated; RRR = 0.37, 95% CI = 0.19, 0.73; *p* < 0.001; *F* = 12.77; Table 2). In a separate sensitivity analysis, we observed that neither people with obesity (but without another high-risk health condition) nor having a high-risk health condition (other than obesity) was associated with being able/willing to quarantine or being willing to vaccinate (compared to not having obesity or any other high-risk health condition); however, experiencing both obesity and a high-risk health condition was associated with a decreased likelihood of having tried to socially distance compared to those who neither had obesity nor another high-risk condition (OR = 0.48, 95% CI = 0.24, 0.96; *p* < 0.035; *F* = 2.89).

## Discussion

More evidence using nationally-representative individual-level data is needed to identify targeted strategies for promoting preventive behaviors among those most vulnerable to COVID-19-related morbidity and mortality. In this study using nationally-representative survey data, we added to the knowledge base by investigating adherence to preventive measures within the population with underlying health conditions and the population of individuals providing caretaking to support those individuals. We also considered how political affiliation relates to adherence to prevention measures. We observed that compared to participants intending to vote for Biden, participants who intended to vote for Trump had a lower likelihood of practicing social-distancing, of self-quarantining, and of intending to be vaccinated. The trend was consistent in the sensitivity analyses restricted to participants with high-risk conditions. In the sensitivity analysis where we did not control for voting preference, individuals with obesity and another high-risk condition were significantly less likely to socially distance compared to individuals with neither obesity nor other high-risk conditions. The finding is unexpected as people with obesity and underlying conditions are more vulnerable to severe COVID-19. Other measures were not independently associated with having a high-risk condition and/or obesity.

Despite variability in the specific measures used as a proxy for partisanship, several nationally representative studies using US data (including ours) suggest that individuals with liberal political leanings (e.g., those that identify as Democrats or Biden voters) are significantly more likely to engage in COVID-19 prevention measures [6, 43, 50]. In our study, we found somewhat stronger effect estimates than others in terms of the relationship between partisanship and willingness to vaccinate. Differences in effect size might be due to differences in precise question wording, the covariates included in each model, the timing of surveys, or sample characteristics.

Our study adds novel insight that can be leveraged to inform outreach strategies and policy targeting the conservative voting base and more broadly. One of our key findings is that individuals with underlying health conditions and a preference to vote for Trump were still significantly less likely to engage in preventative behaviors than those voting for Biden with underlying health conditions. This suggests that messaging about the usefulness of engaging in preventative health behaviors to avoid severe COVID-19 complications may not be reaching (or may not be well-received or perceived as actionable) among vulnerable populations with conservative political affiliations [36–38, 51]. Targeted public health campaigns through community-based partnerships (e.g., with faith-based organizations or with leaders within conservative communities) and policies anchored in behavioral nudges that can boost adherence to preventative measures are needed. People who are vaccine hesitant are more likely to engage in preventive behaviors such as vaccination if messaging is provided by people they trust or admire within their communities [52, 53]. Moreover, there is growing evidence that offering monetary compensation is an effective incentive for COVID-19 vaccination uptake, regardless of political inclination [54]. Although more research is still necessary to define optimal amounts [55], paired with mandates, scale-up of cash incentives and community outreach can become effective targeted policies to improve adherence.

Our findings can also be used to inform preventative messaging for people with obesity and additional high-risk conditions. Results from our study indicate that people with obesity and at least one other high-risk underlying health condition were less likely to socially distance than people who did not have underlying health conditions associated with worse COVID-19 outcomes. This finding offers another opportunity for improved public health messaging, especially if the environments of risk that contribute to underlying health conditions also increase the likelihood of COVID-19 exposure [56, 57]. Further, the normalization of being overweight or obese in US culture, where there is a high prevalence of both, suggests that people may not perceive the health risks of their own health status in general and is it relates to COVID [58]. Notably, messaging that increases stigmatization or shame for those with obesity has been shown to be ineffective [4, 54]; whereas public health messaging focused on positive and empowering ways to increase health have been effective [59, 60]. Public health leaders and others seeking to combat COVID-19 should focus on improving awareness and strengthening systems that allow increased preventative behavior among those with high-risk of severe COVID-19, while being sensitive to the framing of messages to decrease stigma and empower vulnerable populations.

Our study has several strengths and limitations. Strengths include the nationally representative study sample and the ability to use individual-level data to examine associations. One key limitation is the cross-sectional study design which does not allow us to infer causality. Therefore, we are unable to show which preventative measures, or combination of them, are most effective. Future work can extend upon current findings and include a factor analysis to analyze what combination of preventative measures are most effective in preventing deaths. We also base our analysis on participants’ response and cannot be certain if people are behaving as they report or will do so in the future (i.e., social desirability bias may be relevant, especially if individuals with certain voting preferences or certain health statuses are more likely to over or underestimate their preventative behaviors) [61, 62]. Furthermore, data were collected in May 2020; people’s voting preferences, preventative social distancing behavior, or vaccine intentions may have changed since then. Additionally, we cannot examine associations between voter preferences and outcomes among individuals without underlying health conditions due to small numbers of individuals in the sub-analysis (i.e., many cell sizes < 10). Our sample of individuals with underlying health conditions (86%) is also higher than official estimates (over 60%) [63]. Survey weights were estimated based on the full study sample (*n* = 1267), but our analytic sample may not be completely representative of the full study sample. Finally, future research should explore whether alternative underlying factors such as people’s level of altruism and commitment to protecting others against harm could be influencing results [64]. Nevertheless, our study presents novel evidence connecting political preferences, health status, and adherence to COVID-19 preventative behaviors. Public health and community leaders could take the lessons learned to promote adherence to public health prevention efforts [48].

Given the urgent need for improved COVID-19 transmission prevention behaviors, and targeted public health campaigns for high-risk populations and their caretakers, we recommend building on lessons learned from successful prevention strategies and policies that can be applied to the conservative voting base. For example, leveraging community-based partnerships with conservative community organizations and using incentives such as monetary compensation to improve prevention adherence [65]. Increasing access to services and benefits (e.g., paid sick leave) that allow for individuals to engage in preventative health behaviors is also paramount [59, 60]. These actions, supplemented by timely, actionable, and consistent public health messages, could improve adherence to COVID-19 prevention behaviors [61]. If the country is to achieve post-pandemic life through the necessary vaccine-induced herd-immunity and reduce community transmission by strict adherence to mitigation strategies [66], a large scale-up of these community-based initiatives and other policy incentives are needed to establish trust and adherence [7, 51].

## Data Availability

The datasets used and/or analysed during the current study are available from the corresponding author on reasonable request.
